# Bridging Vaccine-Induced HIV-1 Neutralizing and Effector Antibody Responses in Rabbit and Rhesus Macaque Animal Models

**DOI:** 10.1128/JVI.02119-18

**Published:** 2019-05-01

**Authors:** Justin Pollara, Dorothy I. Jones, Tori Huffman, R. Whitney Edwards, Maria Dennis, Shuk Hang Li, Shalini Jha, Derrick Goodman, Amit Kumar, Celia C. LaBranche, David C. Montefiori, Genevieve G. Fouda, Thomas J. Hope, Georgia D. Tomaras, Herman F. Staats, Guido Ferrari, Sallie R. Permar

**Affiliations:** a Department of Surgery, Duke University School of Medicine, Durham, North Carolina, USA; b Human Vaccine Institute, Duke University School of Medicine, Durham, North Carolina, USA; c Department of Pathology, Duke University School of Medicine, Durham, North Carolina, USA; d Department of Cell and Molecular Biology, Feinberg School of Medicine, Northwestern University, Chicago, Illinois, USA; e Department of Pediatrics, Duke University School of Medicine, Durham, North Carolina, USA; Ulm University Medical Center

**Keywords:** ADCC, ADCP, antibody Fc functions, HIV-1 vaccines, rabbit model, rhesus macaques

## Abstract

Studies in animal models are essential prerequisites for clinical trials of candidate HIV vaccines. Small animals, such as rabbits, are used to evaluate promising strategies prior to further immunogenicity and efficacy testing in nonhuman primates. Our goal was to determine how HIV-specific vaccine-elicited antibody responses, epitope specificity, and Fc-mediated functions in the rabbit model can predict those in the rhesus macaque (RM) model. Detailed comparisons of the HIV-1-specific IgG response were performed on serum from rabbits and RM given identical modified vaccinia virus Ankara-prime/gp120-boost immunization regimens. We found that vaccine-induced neutralizing antibody, gp120-binding antibody levels and immunodominant specificities, antibody-dependent cellular phagocytosis of HIV-1 virions, and antibody-dependent cellular cytotoxicity (ADCC) responses against gp120-coated target cells were similar in rabbits and RM. However, we also identified characteristics of humoral immunity that differed across species. ADCC against HIV-infected target cells was elicited in rabbits but not in RM, and we observed differences among subdominantly targeted epitopes. Human Fc receptor binding assays and analysis of antibody-cell interactions indicated that rabbit vaccine-induced antibodies effectively recruited and activated human natural killer cells, while vaccine-elicited RM antibodies were unable to activate either human or RM NK cells. Thus, our data demonstrate that both Fc-independent and Fc-dependent functions of rabbit antibodies can be measured with commonly used *in vitro* assays; however, the ability of immunogenicity studies performed in rabbits to predict responses in RM will vary depending on the particular immune parameter of interest.

**IMPORTANCE** Nonneutralizing antibody functions have been associated with reduced infection risk, or control of virus replication, for HIV-1 and related viruses. It is therefore critical to evaluate development of these responses throughout all stages of preclinical testing. Rabbits are conventionally used to evaluate the ability of vaccine candidates to safely elicit antibodies that bind and neutralize HIV-1. However, it remained unexplored how effectively rabbits model the development of nonneutralizing antibody responses in primates. We administered identical HIV-1 vaccine regimens to rabbits and rhesus macaques and performed detailed comparisons of vaccine-induced antibody responses. We demonstrated that nonneutralizing HIV-specific antibody responses can be studied in the rabbit model and have identified aspects of these responses that are common, and those that are unique, to rabbits and rhesus macaques. Our findings will help determine how to best utilize preclinical rabbit and rhesus macaque models to accelerate HIV vaccine candidate testing in human trials.

## Abstract (precis)

Nonneutralizing antibody functions have been associated with reduced infection risk, or control of virus replication, for HIV-1 and related viruses. It is therefore critical to evaluate development of these responses throughout all stages of preclinical testing. Rabbits are conventionally used to evaluate the ability of vaccine candidates to safely elicit antibodies that bind and neutralize HIV-1. However, it remained unexplored how effectively rabbits model the development of nonneutralizing antibody responses in primates. We administered identical HIV-1 vaccine regimens to rabbits and rhesus macaques and performed detailed comparisons of vaccine-induced antibody responses. We demonstrated that nonneutralizing HIV-specific antibody responses can be studied in the rabbit model and have identified aspects of these responses that are common, and those that are unique, to rabbits and rhesus macaques. Our findings will help determine how to best utilize preclinical rabbit and rhesus macaque models to accelerate HIV vaccine candidate testing in human trials.

## INTRODUCTION

Small-animal model systems are used to evaluate the safety and immunogenicity of candidate immunogens and vaccine regimens and to refine the most promising strategies prior to subsequent preclinical testing in nonhuman primates (NHP). Assessments of vaccine-induced humoral immunity in small-animal studies of candidate human immunodeficiency virus type 1 (HIV-1) vaccines are most often limited to quantifying antibody responses capable of binding to the HIV-1 envelope protein (Env) and of neutralizing the virus by preventing infection of susceptible target cells. These antiviral activities of antibodies are solely dependent on interactions between the antibody and cognate antigen; they do not require other immunoproteins or immune cells. However, there are additional nonneutralizing functions of antibodies that depend on interactions between antibody-antigen immune complexes and immunoproteins or immune effector cells (1–7). Results of immune correlate analyses for the RV144 human clinical trial and preclinical studies conducted in rhesus macaques (RM) have demonstrated that nonneutralizing Fc receptor-dependent antiviral activities of antibodies, including antibody-dependent cell-mediated cytotoxicity (ADCC) and antibody-dependent cellular phagocytosis (ADCP), are important components of immune responses associated with reduced risk of simian immunodeficiency virus (SIV), simian-human immunodeficiency virus (SHIV), or HIV infection or control of viremia (8–17). It is therefore essential to evaluate the development of both neutralizing and nonneutralizing antibody responses throughout all stages of preclinical testing to best inform on the strategies to prioritize for human clinical trials. However, it is not known if small-animal models can effectively predict nonneutralizing antibody responses in humans or RM, which are the most widely used nonhuman primate preclinical model for HIV vaccine testing.

Among commonly used small-animal models, rabbits have several advantages over rodents for studies of humoral immunity (18, 19). Rabbits are intermediate in size between rodents and nonhuman primates, allowing for collection of the larger sample volumes required for in-depth analysis of vaccine-induced immunity. Similar to rodents, rabbits are easy to breed, have short gestation periods, and produce multiple offspring, but the larger size of rabbit kits compared to that of rodent pups makes them more amenable to testing of infant immunization regimens. In addition, prenatal and postnatal transfer of maternal antibodies in rabbits is more similar to that of humans and nonhuman primates (20), making rabbits a more suitable model for evaluating maternal and infant immunization strategies. Rabbits also typically live longer than rodents (21), facilitating testing of vaccine regimens that require multiple boosts and/or long rest intervals between immunizations. Furthermore, rabbits are of particular utility for studies aimed at inducing mucosal immunity through intranasal immunization, as the anatomy of the rabbit nasal cavity is more similar to that of primates (22–24). Finally, rabbits are more phylogenetically similar to humans than rodents (25), and the lack of inbred rabbit strains is likely to more accurately recapitulate the natural diversity of RM and human populations.

In this study, we perform a detailed comparison of humoral responses of rabbits and RM given identical candidate HIV-1 vaccine regimens. Our data demonstrate that the anti-Env IgG and neutralizing antibody responses in rabbits are similar to and predictive of those elicited in RM. Additionally, we use qualified assays that are used to monitor vaccine-induced Fc-dependent antibody activities in human clinical trials to evaluate rabbit and RM serum samples. Similar to the neutralizing antibody responses, we found that ADCC responses directed against gp120-coated target cells are comparable in rabbits and RM. In stark contrast, we find that sera of vaccinated rabbits mediate potent killing of HIV-1-infected cells, while almost no infected cell ADCC is observed for sera from vaccinated RM. We identify species-specific variation in multiple characteristics of the vaccine-induced antibodies that likely contribute to the observed divergence in ADCC activity, including epitope specificities, binding affinities for human Fcγ receptors (FcγR), ability to recruit human innate effector cells, and ADCP activity. Thus, our findings identify both similarities and differences in vaccine-induced humoral immune responses among these two species and suggest further exploration to establish how these models translate to responses induced in human vaccine recipients, as this has important implications for the use of animal models in HIV vaccine development.

## RESULTS

### Antibodies capable of binding HIV-1 gp120 and neutralizing tier 1 viruses were elicited by prime-boost vaccine regimens.

**FIG 1:**
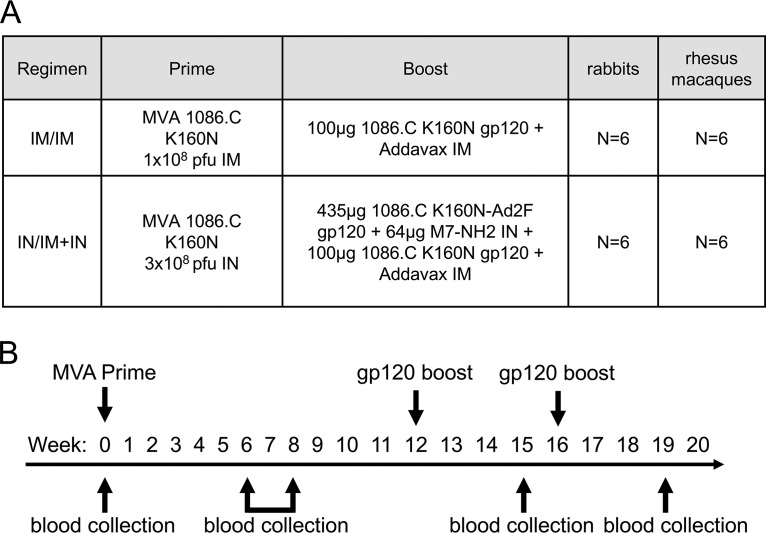
Vaccination groups and study schedule. (A) Systemic (i.m./i.m.) and mucosal (i.n./i.m.+i.n.) vaccine regimens used for immunization of New Zealand White rabbits and rhesus macaques. (B) Schedule of vaccine administration and blood collection.

**FIG 2:**
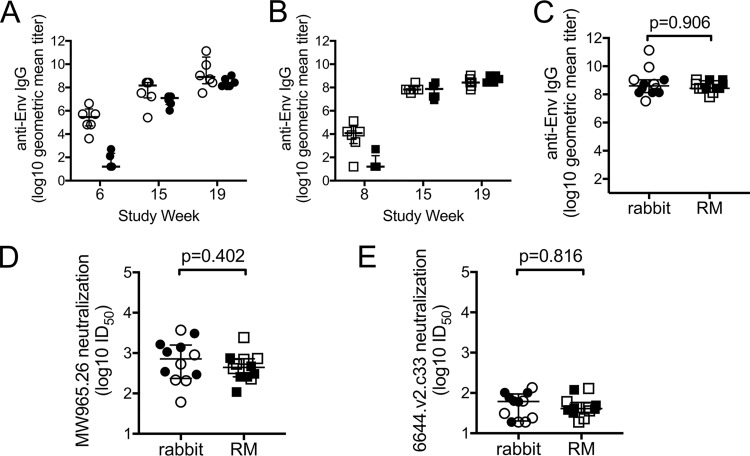
Antibodies capable of binding to gp120 and neutralizing tier 1 viruses were elicited in both rabbits and rhesus macaques (RM). ELISAs were used to measure titers of vaccine-elicited antibodies specific for the 1086.C gp120 protein used as a vaccine immunogen in sera from rabbits (A) and RM (B). (C) No differences (Wilcoxon rank sum test) in anti-Env IgG titers were observed between rabbit and RM sera collected 3 weeks after completion of the vaccine regimens (week 19). Titers of antibodies able to neutralize the tier 1a virus isolate MW965.25 (D) and tier 1b isolate 6644.V2.c33 (E) were similar (Wilcoxon rank sum test) in rabbit and RM sera collected 3 weeks after completion of the vaccine regimens (week 19). Open symbols represent animals that received the systemic i.m./i.m. vaccine regimen, and filled symbols represent animals that received the mucosal i.n./i.m.+i.n. vaccine regimen. Medians are indicated with a horizontal line, and error bars indicate the interquartile range.

Rabbits and rhesus macaques (RM) were immunized with identical prime-boost HIV-1 vaccine regimens (Fig. 1A) according to the same vaccination and sample collection schedules (Fig. 1B). One regimen used conventional intramuscular (i.m.) priming and i.m. boosting (termed i.m./i.m.), while the other was intended to augment mucosal responses through intranasal (i.n.) priming and combined i.m. and i.n. boosting (termed i.n./i.m.+i.n.). We used enzyme-linked immunosorbent assay (ELISA) to measure the kinetics of vaccine-induced gp120-specific antibodies and observed higher responses to the i.m. prime in rabbits (Fig. 2A, week 6, Wilcoxon *P* = 0.004) and RM (Fig. 2B, week 8, Wilcoxon *P* = 0.016) than those observed following i.n. priming. Titers of gp120-specific IgG increased following the first and second protein boost in both groups, and no differences were observed between vaccine groups 3 weeks after completion of the vaccine regimens (week 19, Fig. 2A and B, Wilcoxon *P* = 0.256 and *P* = 0.314, respectively). Due to the similarity between groups at the end of the regimen, we next combined group results as an overall assessment of the vaccine-induced antibody response that we then used to make comparisons across species. Importantly, following completion of the vaccine regimens, we observed no difference in the titers of vaccine-induced gp120-binding antibodies (Fig. 2C) or neutralizing antibody 50% inhibitory dilution (ID_50_) against subtype C tier 1a virus isolate MW965.26 (Fig. 2D) and tier 1b isolate 664.v2.c33 (Fig. 2E) between rabbits and RM. Collectively these data indicate that the vaccines used in our study induced similar gp120-binding and neutralizing antibody responses in rabbits and RM.

### Genetic divergence of immunoglobulin C_H_ regions.

**FIG 3:**
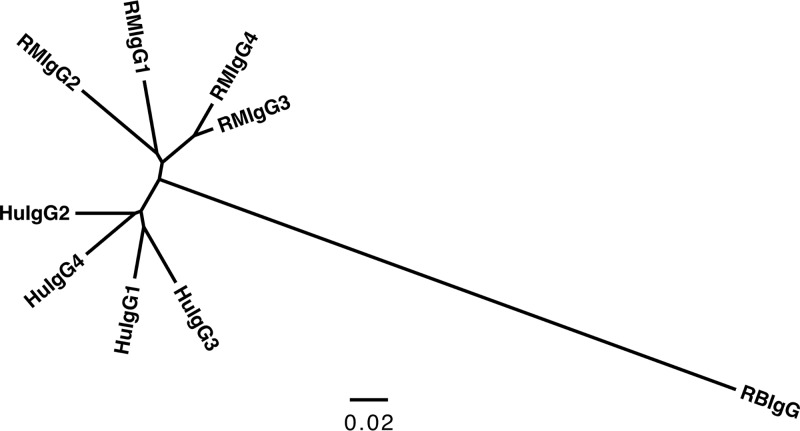
Rabbit IgG constant regions are highly divergent from humans and RM. Genetic comparison of immunoglobulin heavy-chain constant region (C_H_) of rhesus macaque (RM), human (Hu), and rabbit (RB) using a joint radial phylogenetic tree. Scale bar represents the percent sequence dissimilarity as a measure of phylogenetic distance.

The ability of a vaccine-elicited antibody to bind gp120 and to neutralize HIV virions is primarily dependent on the amino acid composition and structure of the Fv region, which establishes the specificity of the paratope and affinity for the cognate epitope (26). In contrast, most nonneutralizing antibody effector functions require both the Fv-dependent formation of an immune complex and interaction of the antibody Fc region with other proteins, such as components of the complement system or specialized Fc receptors (1–7). The Fc region is comprised of the antibody heavy-chain constant domains (C_H_), which in humans and RM define four distinct IgG subclasses (27). On the contrary, rabbits produce only one isotype of IgG (28), and phylogenic analyses indicate that the C_H_ genes of rabbits are highly divergent from the IgG C_H_ genes of humans and RM (Fig. 3). Thus, a concern regarding the use of rabbits as a model to study the immunogenicity of candidate HIV vaccines is that rabbit IgG antibodies may differentially interact with the human Fc receptor-bearing effector cells used in assays that have been developed and optimized to measure the Fc-dependent nonneutralizing activities of human and nonhuman primate antibodies.

### ADCC activity against gp120-coated target cells was elicited in both rabbits and rhesus macaques.

**FIG 4:**
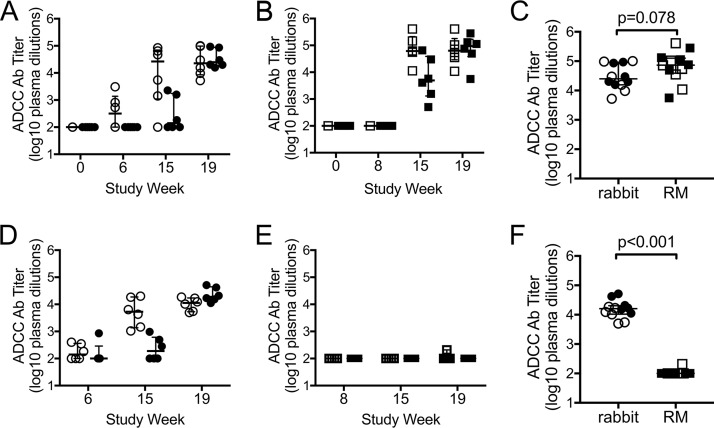
ADCC antibody titers in rabbit and rhesus macaque (RM) immune sera. Titers of ADCC antibody (Ab) directed against target cells coated with the 1086.C gp120 protein used as a vaccine immunogen in rabbit sera (A) and RM sera (B), measured using the ADCC-GTL assay. (C) No difference (Wilcoxon rank sum test) in gp120-specific ADCC Ab titers was observed between rabbit and RM sera collected 3 weeks after completion of the vaccine regimens (week 19). Titers of ADCC Ab directed against target cells infected with HIV-1 1086.C IMC virus in rabbit sera (D) and RM sera (E), measured using the ADCC-Luc assay. (F) Higher infected cell-specific ADCC Ab titers were observed between rabbit and RM sera collected at week 19. Final data were normalized by subtraction of nonspecific activity observe preimmunization (week 0). For all graphs, open symbols represent animals that received the systemic i.m./i.m. vaccine regimen, and filled symbols represent animals that received the mucosal i.n./i.m.+i.n. vaccine regimen. Medians are indicated with a horizontal line, and error bars indicate the interquartile range. *P* values were calculated by Wilcoxon rank sum tests.

We used the ADCC-GranToxiLux (GTL) assay to measure vaccine-induced ADCC responses directed against target cells coated with the recombinant gp120 protein used in the vaccine. This assay uses human peripheral blood as a source of effector cells and has been widely used to measure ADCC responses in both preclinical studies performed in RM and human clinical trials (10, 29–57). ADCC responses against gp120- or gp140-coated target cells have previously been correlated with protection from challenge in preclinical studies, reduced risk of mother-to-child transmission, control of virus replication, and reduced mortality (9, 10, 15, 16, 58, 59). We found that antibodies capable of ADCC activity against gp120-coated target cells were elicited in both rabbits and RM (Fig. 4). In rabbits we were able to detect a postprime response in 3 of 6 animals receiving the i.m./i.m. vaccine regimen, which was not observed in the i.n./i.m.+i.n. group or in any of the vaccinated RM (Fig. 4A and B). However, ADCC antibody titers increased, in both vaccine groups, following the first and second protein boosts in rabbits and RM (Fig. 4A and B). We found no difference in ADCC antibody titers across vaccine groups in either rabbits or RM 3 weeks after administration of the second protein boost (rabbits, Wilcoxon *P* = 0.810; RM, Wilcoxon *P* = 0.688). We therefore combined group data as an overall assessment of the vaccine-induced ADCC responses and compared them across animal models. We found that there was no difference in ADCC antibody titers in rabbit or RM sera after completion of the vaccine regimens (Fig. 4C). We observed the same results in comparisons of maximum ADCC activity (Wilcoxon *P* = 0.885; not shown). Control experiments demonstrated no positive ADCC activity for all rabbit and rhesus samples against uncoated target cells (maximum ADCC activity, <8%; ADCC Ab titer, <1:100; not shown), confirming that the responses are specific to gp120. Collectively, these results indicate that the prime-boost vaccine regimens elicited similar ADCC responses against gp120-coated target cells in these two different animal models.

### ADCC activity against HIV-infected target cells was elicited in rabbits.

Cells coated with recombinant gp120 do not recapitulate all of the natural forms of envelope expressed on the surface of HIV-1-infected cells (60). We therefore used the ADCC-Luciferase (Luc) assay to evaluate vaccine-elicited ADCC activity against target cells infected with an HIV-1 infectious molecular clone (IMC) virus encoding the 1086.C Env protein used for the vaccine immunogen (29, 61). ADCC-Luc assays were performed with the same human peripheral blood mononuclear cells (PBMC) as that used for the ADCC-GTL assay as a source of effector cells. In vaccinated rabbits, we found that antibodies able to direct lysis of HIV-infected cells arose faster in the i.m./i.m. group than in the i.n./i.m.+i.n. group; however, all rabbits in both groups mounted an ADCC response against HIV-infected cells after receiving the second protein boost (Fig. 4D). In stark contrast, only 1 of 12 vaccinated RM elicited an antibody response with ADCC activity against infected cells (Fig. 4E). Concomitantly, we observed significantly higher (Wilcoxon *P* < 0.001) infected cell ADCC antibody titers for rabbit sera (median titer, 16,080) than RM sera (median titer, 100) at completion of the vaccine regimen (Fig. 4F). These data indicate a profound difference in the ADCC response elicited in vaccinated rabbits and RM as measured by the ADCC-Luc assay. This assay is an *in vitro* model that, like many *in vitro* models, is limited in its ability to accurately represent all of the complexities associated with HIV infection, antibody binding, and effector cell recruitment that occur during *in vivo* ADCC responses. Thus, although we cannot speculate from this result what the implication would be *in vivo*, the lack of activity we observed using this model system demonstrates differences in vaccine-induced rabbit and RM antibodies that impact Fc-antibody functions *in vitro*. We next sought to further explore these differences to identify the characteristics of vaccine-induced antibodies that may contribute to the observed species-dependent differential ability of rabbit and RM antibodies to mediate killing of HIV-infected target cells.

### Binding of rabbit and RM antibodies to the surface of HIV-infected cells.

**FIG 5:**
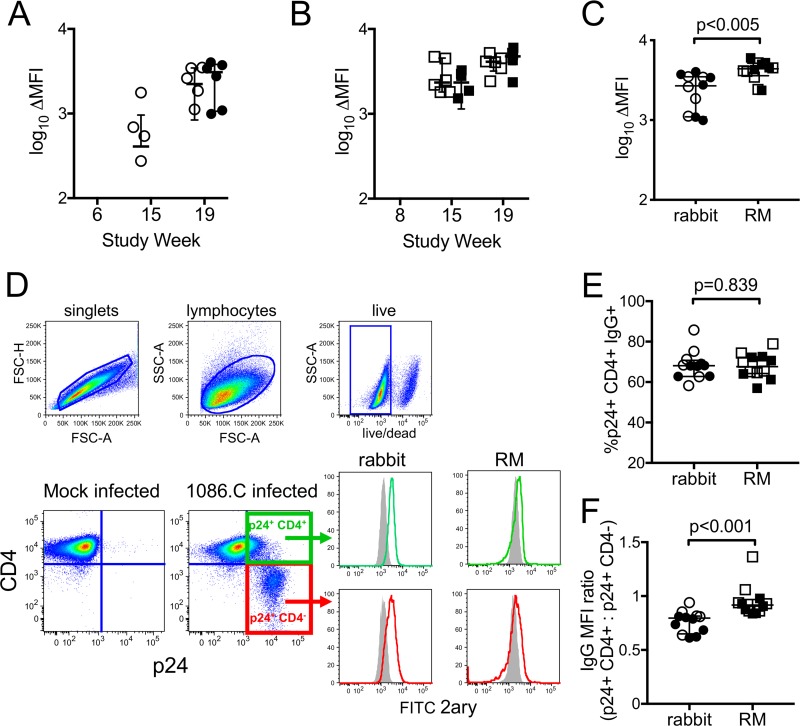
Binding of rabbit and rhesus macaque (RM) antibodies to the surface of HIV-infected cells. Indirect surface staining was used to measure the ability of rabbit (A) and RM (B) immune sera collected 3 weeks after completion of the vaccine regimen (week 19) to bind to the surface of CD4 T cells infected with 1086.C IMC virus. Binding is reported as change in MFI for each week 19 sample compared to week 0 (prevaccination) samples after subtraction of background staining observed in secondary-only wells and mock-infected cells. (C) Comparison of infected cell binding by rabbit sera and RM sera at week 19. (D) Cells infected with HIV-1 1086.C infectious molecular clone virus display two distinct populations of p24^+^ cells: those that retain CD4 on the cell surface (CD4^+^, green gate) and those that no longer express cell surface CD4 (CD4^−^, red gates). Both populations of p24^+^ cells can be recognized by rabbit and RM immune sera (histograms). Gray-filled histograms represent binding by prevaccination serum (week 0), and green or red histograms represent binding by serum collected 3 weeks after final immunization (week 19), as measured by indirect surface staining. A representative example of one rabbit and one RM is shown. FSC, forward scatter; SSC, side scatter. (E) Fraction of the total HIV-infected cells bound by rabbit and RM serum samples collected at completion of the vaccine regimens that retained cell surface CD4. (F) MFI ratio of binding to the populations of infected cells that were CD4^+^ compared to CD4^−^. Open symbols represent animals that received the systemic i.m./i.m. vaccine regimen, and filled symbols represent animals that received the mucosal i.n./i.m.+i.n. vaccine regimen. Medians are indicated with a horizontal line, and error bars indicate the interquartile range. *P* values were calculated by Wilcoxon rank sum tests.

An essential prerequisite of ADCC is that antibodies must form immune complexes with target cells. We used indirect surface staining to evaluate the ability of rabbit and RM vaccine-elicited antibodies to bind the surface of HIV-infected cells. These assays require the use of species-specific secondary antibodies, which can differ in affinities and intrinsic fluorescent properties. Therefore, to account for differences in the properties of the reagents and compare across species, we first normalized the data by subtracting the mean fluorescent intensity (MFI) observed in wells stained with secondary antibody alone. To correct for any nonspecific binding to the human cell line, we also subtracted the MFI observed for mock-infected cells; the final data are reported as the change in measured MFI (ΔMFI) of the fluorescent secondary antibody for staining performed with serum samples collected after vaccination compared to that of samples collected prior to administration of the vaccine. We found that the ability of rabbit antibodies to bind the surface of infected cells (Fig. 5A) paralleled the ADCC activity we previously observed using the ADCC-Luc assay (Fig. 4). Low levels of infected cell binding were observed in the i.m./i.m. group postprime and were increased by subsequent protein boosts. Infected cell-binding antibodies were not detected in the i.n./i.m.+i.n. group until completion of the vaccine regimen, at which time they were present at levels similar to those in the i.m.+i.m. group (Fig. 5A, Wilcoxon *P* = 0.936). Interestingly, antibodies capable of binding to HIV-infected cells were also elicited by both vaccine regimens in immunized RM, and no difference was observed across groups 3 weeks after the final boost (Fig. 5B, Wilcoxon *P* = 0.229). These data indicate that the inability of NHP antibodies to mediate ADCC against infected cells did not result from an absence of antibodies able to recognize the HIV-1 Env expressed on the surface of infected cells. In fact, the change in MFI was higher for rhesus sera than rabbit sera 3 weeks after completion of the vaccine regimens (Fig. 5C).

### Binding specificities of vaccine-elicited antibodies in rabbits and RM.

We next explored whether distinct specificities of antibodies were elicited by the vaccine regimens in rabbits and RM. Antibodies targeting the cluster A region and the coreceptor binding site constitute a class of antibodies that preferentially bind to the CD4-induced (CD4i) conformation of HIV Env. With progression of infection, less CD4 is expressed on the surface of HIV-1-infected cells through the combined effects of the viral Nef and Vpu proteins (62, 63). However, some CD4 is retained on infected cells (64, 65), and two distinct populations of infected (p24^+^) cells can be defined using flow cytometry, one that retains CD4 expression (CD4^+^) and one that downregulates CD4 (CD4^−^) (Fig. 5D). Consistent with previous observations (66), control experiments confirmed that the population of cells that downregulated CD4 was poorly recognized by the CD4i monoclonal antibody (MAb) A32 (A32 binding to p24^+^ CD4^−^, MFI of 639; A32 binding to p24^+^ CD4^+^, MFI of 2,500), suggesting that most of the Env on the surface of these cells was in the closed, non-CD4i conformation. We found that vaccine-induced antibodies in rabbits and RM can recognize both the CD4-downregulated and CD4-expressing cell populations (representative example of rabbit and RM serum in Fig. 5D, histogram panels). We next determined the frequency of p24^+^ CD4^+^ cells recognized by rabbit and RM serum samples collected at completion of the vaccine regimens to provide an indication of the contribution of CD4i antibodies to the total vaccine-elicited polyclonal antibody response. In both rabbits and RM, the majority (frequency, >60%) of antibodies recognized the p24^+^ CD4^+^ population (Fig. 5E), and there was no difference in the frequency of the HIV-1-infected cells recognized by rabbit or RM plasma antibodies. Of note, the ratio of MFI calculated for binding to the p24^+^ CD4^+^and p24^+^ CD4^−^ populations was significantly lower (Wilcoxon *P* < 0.001) for rabbit sera, indicating that more rabbit antibodies bound to the p24^+^ CD4^−^ population than that observed for RM (Fig. 5F). These results demonstrated higher levels of antibody binding to cells that no longer express cell surface CD4 by rabbit vaccine-induced antibodies and suggest that rabbit immune sera more effectively recognize cells that are productively infected with HIV-1.

**FIG 6:**
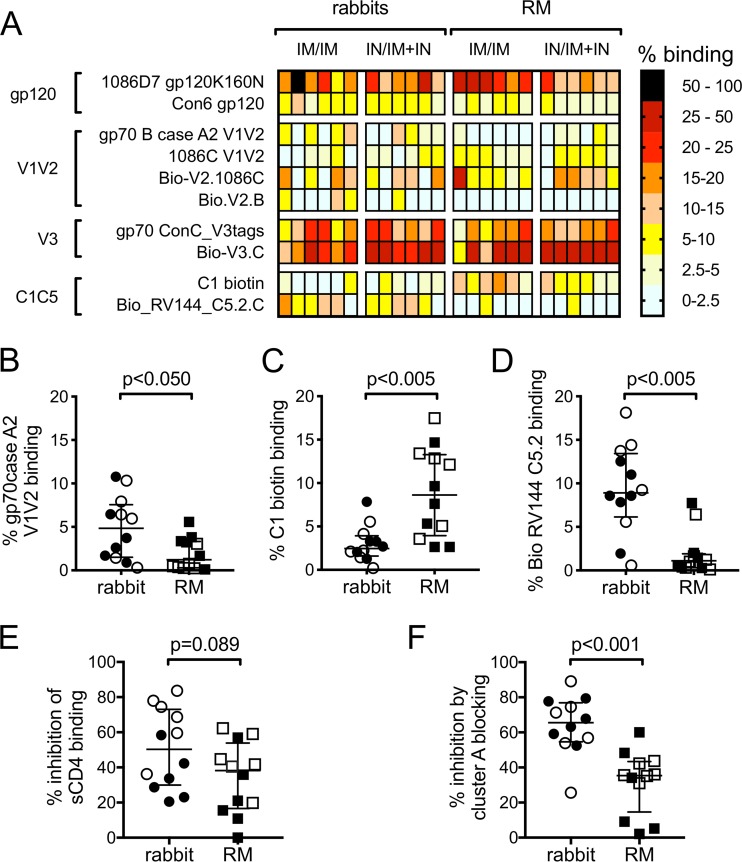
Binding specificities of vaccine-elicited antibodies in rabbits and rhesus macaques (RM). (A) Heat map representing results of HIV-Env-specific IgG binding antibody multiplex assay (BAMA) performed with rabbit and RM serum samples collected 3 weeks after completion of the vaccine regimens (week 19). The percent binding was calculated for each animal by dividing the MFI measured for each antigen by the sum of the total MFIs measured for all antigens. Comparisons of gp70case A2 V1V2 (B), C1 biotin (C), and C5.2 biotin (D) binding antibody responses in rabbit and RM week 19 sera as measured by BAMA. (E) Comparison of CD4 binding site antibody levels in rabbit and RM week 19 sera as determined by soluble CD4 blocking ELISA. (F) Comparison of cluster A antibody levels in rabbit and RM week 19 sera as determined by cluster A blocking assay. In panels B to F, open symbols represent animals that received the systemic i.m./i.m. vaccine regimen, and filled symbols represent animals that received the mucosal i.n./i.m.+i.n. vaccine regimen. Medians are indicated with horizontal lines, and error bars indicate the interquartile ranges. *P* values were calculated by Wilcoxon rank sum tests, with correction for multiple comparisons in panels B to D.

We next used BAMAs to define the specificities of the polyclonal IgG response in rabbits and RM 3 weeks after completion of the vaccine regimens. Because detection reagents for the BAMAs are species specific and differ in fluorescent properties, we calculated the percentage of binding to each antigen to permit direct comparisons across species. This was done on a per-animal basis by dividing the MFI measured for each antigen by the sum of the total MFIs measured for all antigens. As shown by the heat map in Fig. 6A, the V3 region was the dominant target of vaccine-induced antibodies in both rabbits and RM. Species-specific differences were observed among the subdominant responses. The polyclonal antibody response in rabbits included a significantly higher percentage of gp70 case A2 V1V2-binding antibodies (Fig. 6B, Wilcoxon *P* = 0.026), significantly lower percentage of C1-specific antibodies (Fig. 6C, Wilcoxon *P* = 0.004), and significantly higher percentage of C5-specific antibodies than that for RM (Fig. 6D, Wilcoxon *P* = 0.002).

We next evaluated the presence of antibodies specific for the CD4 binding site (CD4bs) using an soluble CD4 (sCD4) blocking ELISA. Control experiments performed with MAbs of known specificities confirmed that the assay was specific for detection of CD4bs antibodies (negative control, MAb 2G12, <5% inhibition of sCD4 binding; positive control, MAb VRC01, >80% inhibition of sCD4 binding). Using this assay, we demonstrated that CD4bs antibodies were present in the circulation of rabbits and RM at completion of the vaccine regimens, and there was no significant difference between the CD4bs antibody response in rabbits and RM (Fig. 6E, Wilcoxon *P* = 0.089).

Antibodies recognizing conformational C1C2 epitopes on the inner domain of gp120, defined as the cluster A region, have been previously identified as potent mediators of ADCC that are commonly generated in response to HIV infection and vaccination (30, 31, 67). To measure the cluster A-specific antibody response elicited in the vaccinated rabbits and RM, we incubated cells infected with HIV-1 1086.C IMC virus with the Fab fragment of MAb A32 and recombinantly produced C11 and CH38 IgA MAbs to bind and block the cluster A region (30, 31, 67). We then performed indirect surface staining as previously described, using our species-specific anti-IgG secondary antibodies that are unable to bind the IgG Fab or IgA antibodies used to block cluster A epitopes. We calculated the percentage of inhibition of binding by rabbit and RM serum samples to the surface of HIV-infected cells by comparing the frequency of cells bound in the absence of cluster A blocking to the frequency of cells bound with cluster A blocking. Blocking with the influenza virus-specific IgA MAb CH65 was used as a negative control, and as expected it had only a modest effect on the binding of rabbit and RM antibodies (5% average inhibition; not shown). We found that significantly more of the total infected cell binding was inhibited upon blocking the cluster A epitope region for assays performed with rabbit samples (65% median inhibition, 55% to 77% interquartile range) compared to RM samples (35% median inhibition, 15% to 43% interquartile range) (Fig. 6F, Wilcoxon *P* < 0.001), indicating that antibodies specific for the cluster A region are a larger component of the antibody response that recognizes HIV-infected cells in vaccinated rabbits than that of RM. Taken collectively with our MFI results showing higher binding of rabbit IgG to cells that have downregulated cell surface CD4 (Fig. 5F), these results suggest that the vaccine-elicited response in rabbits has improved binding to both CD4i and non-CD4i epitopes compared to that for RM.

### Binding of vaccine-elicited antibodies to Fc receptors and interaction with Fc receptor-expressing effector cells.

**FIG 7:**
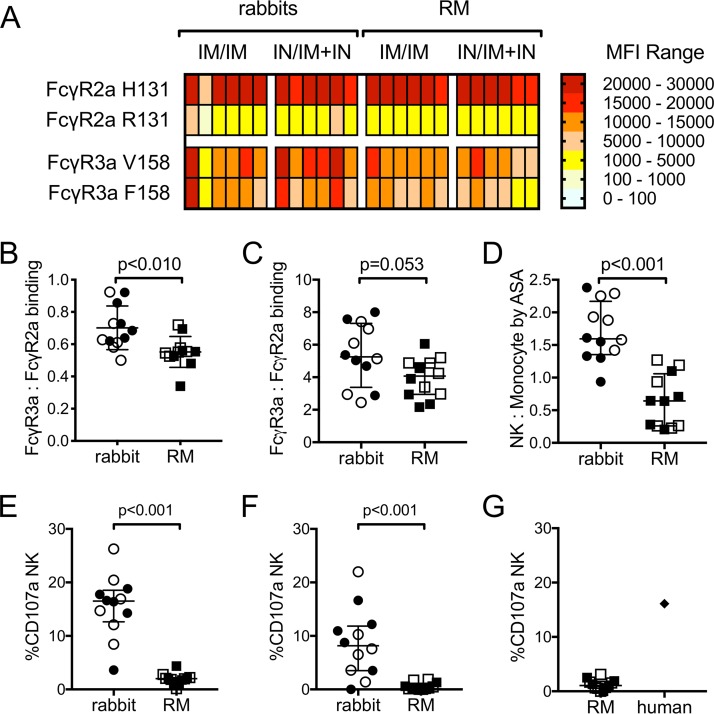
Vaccine-elicited antibodies from rabbits and rhesus macaques (RM) differentially bind to human FcγR and recruit effector cells. (A) Heat map representing results of multiplexed Fc array performed with rabbit and RM serum samples collected 3 weeks after completion of the vaccine regimens (week 19). Assays were performed in duplicate, and MFI values represent the means after blank subtraction. Comparisons of immune complex binding ratios for high-affinity (B) and low-affinity (C) variants of Fcγ3a and Fcγ2a. (D) Area scaling analysis (ASA) was applied to results obtained for week 19 serum samples in the ADCC-GTL assay, and the natural killer (NK) cell-to-monocyte ratio was calculated (see Results). Cell surface expression of CD107a was used as a marker for NK cell degranulation in assays performed using gp120-coated target cells (E) or HIV-infected target cells (F). (G) NK cell degranulation assay performed using PBMC from an unvaccinated RM as effector cells. A human HIV-seropositive plasma sample was used as a positive control. In panels B to G, open circles and squares represent animals that received the systemic i.m./i.m. vaccine regimen, and filled circles and squares represent animals that received the mucosal i.n./i.m.+i.n. vaccine regimen. Medians are indicated with horizontal lines, and error bars indicate the interquartile ranges. *P* values were calculated by Wilcoxon rank sum tests, with correction for multiple comparisons in panels B and C.

Our data suggest that there are differences in the epitope specificities of vaccine-induced antibodies in rabbits and RM, and these antibody Fv region-dependent differences likely contribute to the higher level of HIV-infected cell binding and ADCC activity observed for rabbit antibodies. As ADCC requires engagement of effector cells via the antibody Fc region, it is also probable that rabbit and RM antibodies have differential abilities to engage Fc receptors and initiate an effector cell response. The ADCC assays we performed with gp120-coated targets (Fig. 4A to C) and with HIV-infected targets (Fig. 4D to F) used human PBMCs from the same donor as a source of effector cells. Known single-nucleotide polymorphisms (SNPs) in human Fcγ receptors (FcγR) impact their ability to bind human Fc, resulting in variants with lower or higher relative affinity (5, 7, 68–71). We therefore used PBMC from a donor heterozygous for the common variants of FcγR3a (position 158F, low affinity; 158V, high affinity) and FcγR2a (position 131R, low affinity; 131H, high affinity) to prevent bias related to Fc receptor SNPs in this study. To evaluate the ability of vaccine-induced antibody-antigen immune complexes from rabbits and RM to bind human FcγR, we used a multiplexed human Fc array (72). Immune complexes formed between rabbit and RM serum/plasma samples and the 1086.C gp120 used as the boost immunogen in the vaccine regimens were detected by phycoerythrin (PE)-labeled human Fc receptors, and binding responses were measured as MFI using a Bio-Plex 200 instrument. Binding of gp120-antibody immune complexes to human FcγR2a and FcγR3a variants is shown in Fig. 7A. Interestingly, both rabbit and RM immune complexes were found to bind better to FcγR with the SNPs associated with higher affinity for human antibodies, the 131H variant of FcγR2a and the 158V variant of FcγR3a, indicating some cross-species similarity among rabbits, RM, and humans. Canonical ADCC requires interactions between immune complexes and FcγR3a, while other antibody effector functions, such as phagocytosis, may involve Fcγ2a binding. We therefore next compared the binding of rabbit and RM immune complexes to FcγR3a and FcγR2a to determine if differential binding to human FcγR underlies the observed differences in ADCC activity. We first used MFI ratios to compare binding to the high-affinity variants of FcγR3a (158V) and FcγR2a (131H). As shown in Fig. 7B, MFI ratios were all <1, indicating that both rabbit and RM immune complexes preferentially bind the high-affinity variant of FcγR2a over the high-affinity variant of FcγR3a. However, ratios were significantly higher for rabbit immune complexes than for RM (*P* = 0.008 by Wilcoxon rank sum test), providing evidence of higher levels of interaction with FcγR3a by rabbit immune complexes. We next used MFI ratios to compare binding of immune complexes to the low-affinity variants of FcγR3a (158F) and FcγR2a (131R) (Fig. 7C). We found that MFI ratios for this comparison were all >1, indicating that both rabbit and RM immune complexes preferentially bind to the low-affinity variant of FcγR3a over the low-affinity variant of FcγR2a. The ratios for rabbit immune complexes were higher than those observed for RM immune complexes, indicating a trend toward higher levels of interaction with FcγR3a in rabbits than those in RM (Wilcoxon *P* = 0.053).

Our data from the multiplexed human Fc array suggested that rabbit immune complexes bound better to human FcγR3a than RM immune complexes. We therefore hypothesized that rabbit immune complexes would more effectively recruit human NK cells. To test this, we applied area scaling analysis (ASA) to ADCC-GTL assays performed with gp120-coated target cells (described in the legend to Fig. 4) to discriminate the ability of antibodies to recruit NK cells and monocytes present in the PBMC used as a source of effector cells (73). Using ASA, we calculated the ratio of target cells that interacted with NK cells and monocytes in the presence of serum samples. By this method, ratios of >1 indicate that the antibodies were biased toward recruitment of NK cells, and ratios of <1 indicate that the antibodies were biased toward recruitment of monocytes. As shown in Fig. 7D, vaccine-induced antibodies in rabbits were skewed toward recruitment of human NK cells (median NK/monocyte ratio, 1.6), while the NK cell-to-monocyte ratio was significantly lower for RM antibodies (median, 0.6; *P* < 0.001), indicating preferential recruitment of monocytes. These results are consistent with those of the multiplex human Fc array and provide evidence that vaccine-elicited rabbit antibodies more effectively interact with human FcγR3a and recruit human NK cells than the antibodies from RM.

Finally, we measured the ability of rabbit and RM sera to induce NK cell degranulation using the CD107a assay (74, 75). We found significantly higher levels of CD107a^+^ NK cells in response to rabbit vaccine sera than in RM sera for assays performed with target cells that were either coated with 1086.C gp120 (Fig. 7E) or infected with the 1086.C IMC virus (Fig. 7F). The lack of NK cell degranulation in response to RM immune sera is consistent with results of our FcγR binding assay and ASA analysis and indicates that ineffective interactions between RM sera and FcγR3a on human NK cells are a major determinant of the inability to mediate ADCC against HIV-1-infected targets that we observed in the ADCC-Luc assay (Fig. 4E). We also evaluated the ability of RM sera to induce NK cell degranulation in response to gp120-coated target cells in assays performed using PBMC from an unvaccinated RM as a source of effector cells (Fig. 7G). RM sera did not effectively trigger RM NK degranulation, although RM NK cells did degranulate in response to plasma collected from an HIV-seropositive human included as a positive control.

### ADCP.

**FIG 8:**
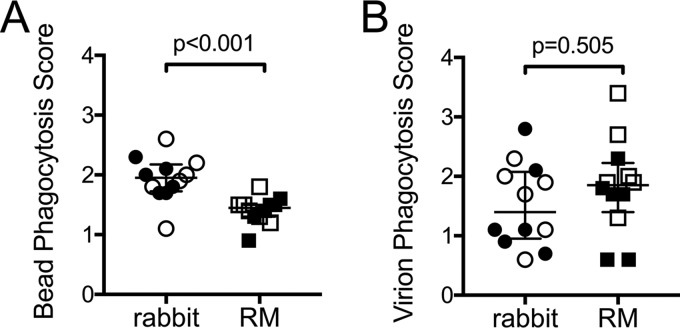
Vaccine-induced antibody-dependent cellular phagocytosis (ADCP) activity in rabbits and rhesus macaques. ADCP activity of rabbit and RM sera collected 3 weeks after completion of the vaccine regimen (week 19) measured in assays performed with primary human monocytes and 1086.C gp120-coated fluorescent beads (A) or HIV-1 1086.C fluorescent virions (B). Phagocytosis scores represent the activity at week 19 after subtracting the week 0 (prevaccination) background activity. Open symbols represent animals that received the systemic i.m./i.m. vaccine regimen, and filled symbols represent animals that received the mucosal i.n./i.m.+i.n. vaccine regimen. Medians are indicated with horizontal lines, and error bars indicate the interquartile ranges. *P* values were calculated by Wilcoxon rank sum tests.

Whereas we found that rabbit vaccine-induced antibodies were better at interacting with human NK cells, our data indicated that RM antibodies more effectively interacted with human FcγR2a and human monocytes. We reasoned that this may result in improved antibody-dependent cellular phagocytosis (ADCP) activity by RM antibodies. To test this, we first measured the phagocytosis of immune complexes formed between 1086.C gp120-conjugated beads and rabbit and RM antibodies using primary monocytes isolated from the same source of human PBMC as that used for ADCC assays as a source of phagocytes. Unexpectedly, we found that significantly more vaccine-induced rabbit immune complexes were phagocytosed than vaccine-induced RM immune complexes (Fig. 8A). We next measured phagocytosis of whole HIV-1 1086.C virions, again using primary human monocytes as phagocytes. As shown in Fig. 8B, there was no difference in the ability of vaccine-induced antibodies from rabbits and RM to mediate virion phagocytosis. These data demonstrate that rabbit and RM immune sera can mediate ADCP; however, the level of activity is impacted by the animal model and the antigen used to produce the immune complex.

## DISCUSSION

Rabbits are an important small-animal model for evaluating the safety and immunogenicity of candidate immunogens and vaccine regimens. Conventionally, the utility of rabbits in HIV vaccine research has been limited to assessment of antibody Fv-dependent functions, such as binding antibody and neutralizing antibody responses. However, evidence from preclinical studies conducted in nonhuman primates and from the RV144 clinical trial have implicated Fc-dependent antibody functions as components of the immune responses associated with reduced risk of SIV, SHIV, and HIV infection or control of viremia (8–17), making it critical to monitor the elicitation of antibodies with these functions throughout preclinical vaccine development. Unfortunately, there has been a gap in knowledge regarding how effectively small-animal models can predict Fc-dependent antibody responses in primates or humans. Here, we performed a detailed comparison of HIV-1 vaccine-induced humoral responses in rabbits and RM, which are the most relevant and widely used nonhuman primate model for HIV vaccine research. Rabbits and RM were administered identical vaccine regimens, and blood samples were collected according to matched schedules. All immunogenicity assessments were conducted in parallel, using the same methodologies and reagents wherever possible to minimize assay variability and, thus, maximize our ability to identify aspects of the humoral response that are unique to each particular animal model. As expected, we found that anti-Env IgG and neutralizing antibody responses were induced with similar kinetics and magnitude in vaccinated rabbits and RM. We also found no differences in ADCC directed against gp120-coated target cells. However, we found a dramatic difference in ADCC directed against HIV-infected cells, with rabbit sera capable of mediating potent cell killing, while almost no ADCC was observed with sera from vaccinated RM. To identify the specific characteristics of vaccine-induced antibodies underlying the species-dependent differential ability to mediate ADCC of HIV-infected targets, we defined the infected cell binding, epitope specificity, FcγR binding, effector cell recruitment, and ADCP activity of rabbit and RM immune sera.

ADCC activity against HIV-1-infected cells requires antibodies capable of binding to the forms of Env present on the cell surface. The vaccine regimens used in this study induced infected cell-binding antibodies both in rabbits and RM. We therefore hypothesized that the ability to kill infected cells by rabbit antibodies was related to differences in the Env epitopes recognized by vaccine-induced antibodies. We identified the V3 region as the dominant target of vaccine-induced IgG in both animal models. Species-specific differences were found among the subdominantly targeted epitope regions, including higher levels of V1V2 and C5 binding antibodies in rabbits and higher levels of C1 antibodies in RM. In addition, antibodies specific for the CD4i cluster A region, a previously identified target of potent ADCC antibodies, were more prevalent in vaccinated rabbits. However, we also found higher levels of antibody binding to cells that no longer express cell surface CD4 by rabbit antibodies, indicating that rabbit immune sera are also more effective at targeting non-CD4i epitopes on the surface of infected cells than RM sera. Although likely contributing factors, the differences in epitope specificities are not sufficient to explain the nearly complete lack of ADCC activity against infected cells in vaccinated RM when this vaccine-elicited IgG response in rabbits was readily detectable. We therefore next investigated the interaction of rabbit and RM sera with human FcγRs and FcγR-expressing innate effector cells.

For the ADCC assays performed in this study, we used human PBMC from a donor heterozygous for the common variants of FcγR2a and FcγR3a as the source of effector cells (5, 7, 68–71). Using BAMA Fc arrays, we found higher levels of FcγR3a binding by immunocomplexes formed with rabbit IgG than RM IgG. Consistent with this observation, application of ASA to the results of the ADCC-GTL assay (73) demonstrated that rabbit serum preferentially recruited human NK cells. Moreover, we demonstrated that rabbit immune sera were able to induce a significantly higher frequency of NK cell CD107a degranulation (74) than RM sera. The lack of NK cell degranulation in response to RM immune sera suggests that although the RM antibodies induced by this vaccine regimen are able to form immune complexes and interact with human Fcγ receptors, they fail to fully recruit an effective NK cell response. Interestingly, we also demonstrated that RM immune sera was also unable to trigger degranulation of RM NK cells present in RM PBMC. This suggests that the deficiency is at the level of RM vaccine-induced IgG and not an artifact of the cross-species interactions required when human PBMC are used as effectors. The inability of the vaccinated RM sera to induce NK cell degranulation explains the lack of ability to mediate ADCC against HIV-1-infected cells, as observed in the ADCC-Luc assay. Additional work will be required to determine why rabbit, but not RM, vaccine-elicited IgG responses can trigger NK degranulation. One possible contributing factor is species-dependent differences in the level of fucosylated IgG core oligosaccharides (76).

Although NK cells also contribute to the activity observed against gp120-coated target cells in the ADCC-GTL assay, we have previously demonstrated that this assay also measures non-ADCC interactions between antibodies and monocytes (73). Therefore, we next explored possible differences in monocyte ADCP by rabbit and RM immune sera. Because our data suggested that rabbit antibodies did not interact with human monocytes as effectively as RM antibodies, we expected that RM sera would mediate higher levels of ADCP. In fact, higher levels of ADCP were observed with rabbit sera in assays performed using gp120-coated beads as targets for immune complex formation. Moreover, we observed no difference in ADCP activity of rabbit and RM sera when using fluorescent HIV-1 1086.C virions as a target for immune complexes and primary human monocytes as a source of phagocytes. These data demonstrate that interplay between antibody, antigen, and phagocytes affects ADCP activity. An additional complication that may affect interpretation of these results is that human monocytes express cell surface FcαRI and can use this receptor for phagocytosis of antigen-IgA immune complexes (77). Our FcR BAMA arrays did not include FcαRI, and we therefore cannot rule out possible interactions between rabbit or RM IgA and human FcαRI. Additional studies will be required to define the potential involvement of vaccine-elicited rabbit and RM IgA in the total ADCP activity observed with our assays.

Recent studies have described the complexity of HIV ADCC and potential caveats associated with attempts to measure it *in vitro* (66, 78). The assays and target cells we used in this study are those commonly used for immune monitoring of preclinical and clinical trials, have been correlated with relevant biological outcomes, and are expected to be capable of detecting responses that recognize virus at different stages of infection (10, 15, 16, 29–59). It is important to note, however, that they are only *in vitro* model systems, and as such they are limited in the degree to which they accurately represent ADCC *in vivo*. Target cells coated with HIV Env gp120 likely represent a variety of potential target cells *in vivo*, including both nonpriority targets, such as shed-Env-coated bystander cells (78), and the priority target of cells that are interacting with HIV virions at the time of virus entry, as evidenced by previously described correlations with assays performed using whole-virion coating (39, 79). In contrast, HIV-infected cells likely represent target cells during the time of virus assembly and release. Our IMC virus included a luciferase reporter gene, which has been described to interfere with expression of the viral Nef protein, resulting in inefficient downregulation of CD4 from the cell surface (66). To address this concern, we show the extent of CD4 downregulation in Fig. 5D and confirmed reduced binding of the CD4i-specific MAb A32 to the population of cells downregulated for CD4, suggesting a large portion of the HIV Env trimer is in the closed, non-CD4i conformation on these cells, as previously demonstrated (66). It is also important to note that the functionality of Nef differs across virus isolates (80), as does the intrinsic closed or open status of Env trimers (81, 82), making it unlikely that single-virus infection, assayed at a single time postinfection, will reflect the complexity of HIV infections *in vivo*. Our combined use of gp120-coated target cells and infected target cells that present both open and closed HIV envelope on the cell surface is expected to provide a broad assessment of possible ADCC targets, because it facilitates detection of antibody responses recognizing different putative envelope conformations exposed from early after attachment through virus budding. Given the caveats described for the different types of target cells used, as well as those related to performing assays with cross-species effector cells, we would not take the ADCC activity or lack thereof measured by our *in vitro* assays as evidence of the ability of these vaccine regimens to elicit protective ADCC responses *in vivo*. For RM, this would require further study that includes use of appropriate SHIVs and autologous primary target and effector cells. However, through use of these *in vitro* models that are widely used in preclinical studies and human clinical trials, we identified species-specific differences in vaccine-elicited antibodies that impacted the ADCC activity as measured, such as ability or inability to induce NK cell degranulation. Ultimately, determination of which types of target cells and ADCC assays best model the complexity of cell-to-cell interactions that take place at the tissue level *in vivo* will depend on the ability or inability to correlate with efficacy in current and future preclinical studies and human clinical trials.

Through detailed comparison of humoral responses of rabbits and RM given identical candidate HIV-1 vaccine regimens, we have identified characteristics of the humoral response that are similar in these two animal models, including kinetics and magnitude of binding antibodies, levels of neutralizing antibodies, ADCC activity against gp120-coated cells, immunodominant epitope specificities, and ADCP of HIV-1 virions. However, we also identified characteristics of the humoral response that differ across species, including ADCC activity against infected cells, subdominant epitope specificities, affinity for FcγR and recruitment of innate effector cells, and ADCP of gp120-coated beads. Thus, our data demonstrate that the ability of immunogenicity studies performed in the rabbit model to predict responses in nonhuman primates will vary depending on the particular immune parameter of interest, as recently described (83). Importantly, our study has also demonstrated that rabbit vaccine-elicited IgG interacts effectively with human Fcγ receptors and innate effector cells, despite the genetic divergence between rabbit C_H_ genes and primate C_H_ genes. These results are consistent with recent studies from our group and others that evaluated vaccine-induced HIV-1-specific ADCC in studies performed in rabbits (84–86).

Although direct comparison of identical vaccine regimens is a strength of our study, it also limits how broadly applicable our conclusions can be applied to other vaccine platforms. Moreover, our study was also limited in that we were unable to compare humoral responses in our animal models to those observed in human clinical trials. While preclinical HIV-1 vaccine studies performed in RM and human clinical trials have been shown to recapitulate safety and immunogenicity results (45, 87–92), the rabbit model has not been compared in similar detail. However, translation of studies performed in the rabbit model to studies performed in RM and humans aided in the development of next-generation anthrax vaccine regimens (93–96). Thus, it will be interesting in future studies to determine how effectively vaccine-induced HIV-1 immunity in rabbits translates directly to the results of human clinical studies.

In summary, we have demonstrated both Fc-independent and Fc-dependent antibody responses can be studied in the rabbit small-animal vaccine model. We have compared these responses to those elicited in vaccinated RM and have identified aspects of the humoral response that are common, and those that are unique, to each animal species. Future studies bridging these two model systems with results of human clinical trials will help to determine how to best utilize small-animal models to accelerate development of human clinical trials.

## MATERIALS AND METHODS

### Animal models and vaccine regimens.

New Zealand White rabbits were obtained from Robinson Services (female rabbits; Mocksville, NC) or Covance (male rabbits; Princeton, NJ) and housed at Duke University School of Medicine. Twelve rhesus macaques between 1 and 3 years of age were obtained from the New England Primate Research Center and housed at the same facility. Animals were maintained according to the guidelines of the National Institutes of Health, Harvard Medical School, and Duke University. All studies were approved by the appropriate Institutional Animal Care and Use Committee. Animals were assigned to the vaccine groups indicated in Fig. 1A. Rabbits were assigned sequentially as obtained from the supplier; RM were stratified by age and weight to prevent age or weight bias in the vaccine groups. Immunizations and sample collection were performed according to the indicated schedule (Fig. 1B).

### Measurement of HIV-specific binding antibody by ELISA.

ELISAs were performed as described previously (97). Plates were coated with the vaccine boost protein (1086.C K160N gp120 [98]) at 2 µg/ml. Serum samples were tested using serial 2-fold dilutions starting at 1:32. Rabbit antibodies were detected using alkaline phosphatase-conjugated goat anti-rabbit IgG (catalog number 4030-04; Southern Biotech, Birmingham, AL). RM antibodies were detected using anti-NHP IgG (catalog number 617-105-012; Rockland Immunochemicals Inc., Limerick, PA). Endpoint titers were the last immune sample dilution that was 3-fold higher than the dilution-matched preimmune sample. Immune sample dilutions that were less than 3-fold higher than the naive sample at the starting dilution were reported as one log_2_ dilution lower than the starting dilution.

### Neutralization assays.

Neutralization was measured as the ability of serum samples to reduce virus infection of TZM-bl cells as previously described (99). Briefly, serum was incubated with a subtype C tier 1a isolate (MW965) or tier 1b isolate (6644.v2.c33) pseudovirus for 45 min at 37°C (100, 101). TZM-bl cells were added and allowed to incubate for 48 h. A luciferase reagent (Bright-Glo; Promega, Madison, WI) was added, and luminescence was measured. Results were reported as the 50% inhibitory dilution (ID_50_), which is the dilution of serum resulting in 50% reduction in luminescence compared to that of virus control wells.

### IgG phylogenic analysis.

IgG heavy-chain constant region sequences were downloaded from the public domain. The sequences were aligned by CLUSTAL O algorithm using the Seaview program (102). A neighbor-joining tree for the aligned sequences was constructed using the Kimura 2-parameter model.

### ADCC-GTL assay.

The ADCC-GTL assay was performed as previously described (73, 103). Target cells were a clonal isolate of the CEM.NKR_CCR5_ CD4^+^ T cell line (NIH AIDS Reagent Program, Division of AIDS, NIAID, NIH; from Alexandra Trkola [104]) coated with 1086.C K160N gp120 protein. Effector cells were PBMC obtained from an HIV-seronegative donor heterozygous for FcγR3A at position 158 (158F/V) and for FcγR2A at position 131 (131H/R) (5, 68, 69). PBMC were obtained by leukapheresis to collect enough cells for completion of the study with a single donation, minimizing any potential for variability in the effector cell populations to influence the study outcome. PBMC were used at an effector cell/target cell ratio of 30:1. Serum samples were tested after 4-fold serial dilutions starting at 1:100. Data were reported as the maximum proportion of cells positive for proteolytically active granzyme B (GzB) out of the total viable target cell population (maximum %GzB activity) after subtracting the background activity observed in wells containing effector and target cells in the absence of plasma. ADCC endpoint titers were determined by interpolating the last serum dilution above the previously established positive cutoff for this assay (8% GzB activity) using GraphPad Prism, version 7.0b, software (GraphPad Software, Inc., La Jolla, CA) and were reported as reciprocal dilution. ASA of the GzB^+^ cells was used to evaluate antibody recruitment of NK cells and monocytes as previously described (73).

### ADCC-Luc assay.

ADCC activity directed against HIV-infected target cells was determined by the ADCC-Luc assay as previously described (29, 73). CEM.NKR_CCR5_ target cells were infected with HIV-1 1086.C infectious molecular clone virus encoding *Renilla* luciferase (61) for 48 h and then incubated with PBMC effector cells (30:1 effector cell/target cell ratio) and serum dilutions in half-area opaque flat-bottom plates (Corning Life Sciences, Corning, NY), in duplicate wells, for 6 h at 37°C and 5% CO_2_. ADCC activity, reported as percent specific killing, was calculated from the change in relative light units (RLU) (ViviRen luciferase assay; Promega) resulting from the loss of intact target cells in wells containing effector cells, target cells, and serum samples compared to RLU in control wells containing target cells and effector cells alone according to the following formula: percent specific killing = [(number of RLU of target and effector well − number of RLU of test well)/number of RLU of target and effector well] × 100. ADCC endpoint titers were determined by interpolating the last serum dilution above the positive cutoff for this assay (15% specific killing) using GraphPad Prism, version 7.0b, software after subtracting the background activity observed for matched prevaccination samples and were reported as reciprocal dilution.

### Infected cell antibody binding assay.

Indirect surface staining was used to measure the ability of serum antibodies to bind HIV-1 Env expressed on surfaces of infected cells using methods similar to those previously described (10). CEM.NKR_CCR5_ cells were mock infected or infected with an HIV-1 infectious molecular clone virus expressing the 1086.C Env protein for 48 h. The cells were then washed and incubated with a 1:100 dilution of plasma samples for 2 h at 37°C and then stained with Live/Dead Aqua dead cell stain (Thermo Fisher Scientific, Waltham, MA) to exclude dead cells from analysis. For experiments where CD4 dependence was determined, the cells were stained with anti-human peridinin chlorophyll protein-Cy5.5 conjugated CD4 antibody (clone OKT4; eBioscience, Thermo Fisher Scientific) for 30 min at room temperature. For all experiments, cells were next washed and then permeabilized with Cytofix/Cytoperm solution (BD Biosciences, San Jose, CA) prior to staining with RD1-conjugated anti-p24 MAb KC57 (Beckman Coulter, Inc., Indianapolis, IN) and fluorescein isothiocyanate (FITC)-conjugated goat anti-rhesus IgG (H+L) polyclonal antiserum or anti-rabbit IgG (H+L) (both from Southern Biotech). Cells positive for serum antibody binding were defined as viable, p24 positive, and FITC positive. Final results were reported as the change in FITC median fluorescent intensity (MFI) for postvaccination samples compared to that of the prevaccination sample, gated on the live, infected cell population (p24-positive cells) after subtraction of the MFI observed for cells stained with secondary antibody alone and mock-infected cells.

### HIV Env-specific IgG binding antibody multiplex assay.

HIV Env-specific antigens were conjugated to polystyrene beads (Bio-Rad), and the coupled beads were used to approximate IgG binding in rabbit and rhesus plasma through a binding antibody multiplex assay (BAMA), as previously described (105). The following panel of antigens was tested: gp70 B case A2 V1V2, 1086.C V1V2, gp70 ConC_V3tags, Con6 gp120/B (106), and 1086.C K160N gp120. The following Env peptides were used: Biotin (Bio) V2.1086C (Bio-KKKTELKDKKHKVHALFYKLDVVP), Bio V3.C (Bio-KKKNNTRKSIRIGPGQTFYATGDIIGDIRQAHC), Bio V2.B (Bio-KKKTSIRDKVQKEYALFYKLDVVP), C1 Biotin (Bio-KKKMQEDVISLWDQSLKPCVKLTPLCV), and Bio_RV144_C5.2.C (Bio-KKKSELYKYKVVEIKPLGIAPTKAKRRVVEREKRAV). MulV gp70 His6 and blank beads were also included as a negative-control antigen to account for nonspecific binding. The antigen-conjugated beads were incubated with rabbit and rhesus plasma at a 1:500 dilution. RIVIG and BIVIG (rhesus/bunny immunodeficiency virus immune globulin) were used as positive controls for the rhesus plasma and rabbit plasma, respectively. IgG binding in rhesus plasma was detected with PE-conjugated mouse anti-monkey IgG antibody (Southern Biotech) at 4 µg/ml. Similarly, IgG binding in rabbit plasma was detected with a phycoerythrin (PE)-conjugated goat anti-rabbit IgG detection antibody (Southern Biotech) at 2 μg/ml. Assays were read on a Bio-Plex 200 instrument (Bio-Rad Laboratories, Inc., Hercules, CA), and binding results were expressed in MFI units. To control for background binding, the MFI of beads in wells that did not contain sample was subtracted from sample-specific MFIs within respective assay plates. An HIV Env-specific antibody response was considered positive if it had MFI values above the lower detection limit of 100 MFI. To ensure consistency between assays, 50% effective concentration and maximum MFI values of the positive controls (RIVIG and BIVIG) were tracked by Levey-Jennings charts. To normalize our rabbit and RM data, collected using species-specific detection reagents, we calculated the percentage of binding to each antigen by dividing the MFI measured for each antigen by the sum of the total MFIs measured for all antigens.

### Soluble CD4 blocking ELISA.

For CD4 blocking ELISAs, 384-well plates (Corning Life Sciences) were coated with 1086.C K160N gp120 at 30 ng/well. The plates were blocked with assay diluent (phosphate-buffered saline containing 4% whey, 15% normal goat serum, and 0.5% Tween 20) for 1 h at room temperature. MAbs or plasma samples next were added and incubated for 1 h, and then sCD4 (human soluble CD4 recombinant protein from progenics; NIH AIDS Reagent Program, Division of AIDS, NIAID, NIH) was added at 640 ng/ml. sCD4 binding was detected using a biotinylated human anti-CD4 antibody (Thermo Fisher Scientific, San Diego, CA), followed by horseradish peroxidase-conjugated Streptavidin. Percent sCD4 binding inhibition was calculated as 100 – (average of serum duplicate optical density/average of duplicate negative-control optical density) × 100.

### Cluster A blocking.

CEM.NKR_CCR5_ cells were infected with HIV-1 1086.C IMC as described above. The cells were then washed and incubated with a cluster A antibody blocking cocktail (10 μg/ml each of MAb A32 IgG Fab, MAb C11 IgA, and MAb CH38 IgA [30, 31, 67]) for 15 min at 37°C. Rabbit and RM plasma/serum samples were then added at 1:100 dilution, and indirect surface staining was performed as described above. Infected cells alone or cells incubated with the influenza virus-specific MAb CH65 IgA were used as controls to confirm the specificity of the blocking. The percent inhibition by cluster A blocking was calculated by dividing the frequency of cells bound in the absence of cluster A blocking by the frequency of cells bound with cluster A blocking. Blocking with the influenza virus-specific IgA MAb CH65 was used as a negative control and, as expected, had only a modest effect on the binding of rabbit and RM antibodies (5% average inhibition).

### Multiplexed Fc array.

Multiplexed Fc array was used to evaluate FcγR binding of antigen-specific antibodies. Preparation and biotinylation of AVI-tagged FcγRs was done using previously described methods (72), except that the proteins were expressed in 293-F cells (Thermo Fisher Scientific). All plasma samples were tested at a 1:50 dilution against 25 μg of 1086.C K160N gp120, coupled to 5 × 10^6^ carboxylated fluorescent beads (Bio-Rad Laboratories). Antigen-coupled beads and diluted plasma samples were incubated for 30 min, and then FcγR binding was measured. Biotinylated FcγRs were combined with Streptavidin PE using a 4:1 ratio and mixed for at least 10 min before adding to the wells. FcγR binding was detected with the biotinylated FcγR-Streptavidin PE mixture at 4 μg/ml after 2.5 h of incubation. Binding responses were acquired using a Bio-Plex 200 instrument (Bio-Rad Laboratories). Purified IgG from pooled plasma of HIV-1-vaccinated rhesus macaques and rabbits (RIVIG and BIVIG, respectively) was used as a positive control. Fc binding responses were expressed as MFI values. All MFI values were blank subtracted and represent the means from assay duplicates. Binding responses above 100 MFI were considered positive.

### CD107a degranulation assay.

Cell surface expression of CD107a was used as a marker for NK cell degranulation (74, 107). CEM.NKR_CCR5_ target cells were either coated with 1086.C K160N gp120 protein or infected with HIV-1 1086.C infectious molecular clone virus (48 h of infection) and then plated with human PBMC effector cells and 1:50 dilutions of sera in V-bottom plates (Corning Life Sciences) in duplicate wells (1 × 10^4^ target cells and 3 × 10^5^ PBMC per well). Brefeldin A (GolgiPlug; 1 μl/ml; BD Biosciences), monensin (GolgiStop; 4 μl/6 ml; BD Biosciences), and CD107a-FITC (clone H4A3; BD Biosciences) were added to each well, and the plates were incubated for 6 h at 37°C in a humidified 5% CO_2_ incubator. Plates were then washed and stained with a viability marker and fluorescently conjugated antibodies (PE-Cy5-CD14, clone Tuk4; Thermo Fisher Scientific; PE-Cy7-CD56, clone NCAM16.2; BD Biosciences; PacificBlue-CD16, clone 3G8; BD Biosciences; PE-Cy5-CD19, clone SJ25-C1; Thermo Fisher Scientific; allophycocyanin [APC]-Cy7-CD3, clone SP34-2, and APC-H7-CD4, clone SK3; BD Biosciences) using standard techniques. Data analysis was performed using FlowJo software (v9.9.6). Data are reported as the percentage of CD107a-positive live NK cells at week 19 (singlets, lymphocytes, live, CD3^−^, CD14^−^, CD19^−^, CD4^−^, CD56^+^, and/or CD16^+^ and CD107a^+^) after subtraction of the percent CD107a^+^ live NK cells in the preimmune samples. For assays performed with RM PBMC effectors, we used the following antibodies: PE-Cy5-CD107a, clone H4A3; PacificBlue-CD3, clone SP34; AF700-CD20, clone 2H7; APC-H7-CD8, clone SK1 (all from BD Biosciences); and PE-NKG2A/C, clone Z199 (from Beckman-Coulter). To identify the population of interest, the following gating strategy was applied: singlets, lymphocytes, live, CD3^−^, CD20^−^, CD8^+^, NKG2A/C^+^, and CD107a^+^.

### ADCP assays.

ADCP of 1086.C K160N gp120-coated fluorescent beads or fluorescently labeled HIV-1 1086.C virions (108, 109) was measured as previously described (77, 110). Briefly, 9 × 10^5^ gp120-coated beads or 10 μl of fluorescent HIV-1 1086.C was mixed with 10 μl of serum for 2 h at 37°C to permit formation of immunocomplexes prior to addition of primary human monocytes that were isolated from PBMC by negative selection (human pan-monocyte isolation kit [Miltenyi Biotec, GmbH] used at 1 × 10^4^ cells per well). PBMC used for monocyte isolation were from the same donor as that used for ADCC assays. Spinoculation, incubation, CD4 blocking, washes, data acquisition, and data analysis were performed as previously described (77). ADCP activity was presented as the phagocytosis score, calculated by the percentage of cells positive times the MFI, normalized to the corresponding result for the no-antibody control. Final data represent the phagocytosis score for sera collected at completion of the regimen after subtracting the score for matched preimmunization samples; thus, no positive cutoff was applied. Assays were performed in duplicate for each animal.

### Statistical analysis.

Wilcoxon rank sum tests were used to assess if the two different vaccine regimen groups had different immunologic responses within each species and to compare immunologic responses across the species after combining the two different vaccine treatment groups to make an overall assessment of vaccine-induced response. *P* values of less than 0.05 were considered significant. The Benjamini and Hochberg false discovery rate (FDR) method was used to correct for multiple testing across responses to maintain an adjusted *P* value of 0.05 (111). Antibody titers obtained from ELISA as well as neutralization and ADCC assays were log transformed for all analyses. Statistical analysis was performed using SAS software (SAS Institute, Inc., Cary, NC).
